# Experimental Tumor Induction and Evaluation of Its Treatment in the Chicken Embryo Chorioallantoic Membrane Model: A Systematic Review

**DOI:** 10.3390/ijms25020837

**Published:** 2024-01-09

**Authors:** Cristina Mesas, Maria Angeles Chico, Kevin Doello, Patricia Lara, Javier Moreno, Consolación Melguizo, Gloria Perazzoli, Jose Prados

**Affiliations:** 1 Institute of Biopathology and Regenerative Medicine (IBIMER), Biomedical Research Center (CIBM), 18100 Granada, Spain; cristinam@correo.ugr.es (C.M.); patricialara@correo.ugr.es (P.L.); javiermperez99@correo.ugr.es (J.M.); jprados@ugr.es (J.P.); 2 Instituto de Investigación Biosanitaria de Granada (ibs.GRANADA), 18012 Granada, Spain; mchico@ugr.es (M.A.C.); kdoello@correo.ugr.es (K.D.); 3 Department of Anatomy and Embryology, University of Granada, 18071 Granada, Spain; 4 Service of Medical Oncology, Hospital Virgen de las Nieves, 18014 Granada, Spain

**Keywords:** CAM, tumor, xenograft, in ovo, ex ovo

## Abstract

The chorioallantoic membrane (CAM) model, generated during avian development, can be used in cancer research as an alternative in vivo model to perform tumorigenesis in ovo due to advantages such as simplicity, low cost, rapid growth, and being naturally immunodeficient. The aim of this systematic review has been to compile and analyze all studies that use the CAM assay as a tumor induction model. For that, a systematic search was carried out in four different databases: PubMed, Scopus, Cochrane, and WOS. After eliminating duplicates and following the established inclusion and exclusion criteria, a total of 74 articles were included. Of these, 62% use the in ovo technique, 13% use the ex ovo technique, 9% study the formation of metastasis, and 16% induce tumors from patient biopsies. Regarding the methodology followed, the main species used is chicken (95%), although some studies use quail eggs (4%), and one article uses ostrich eggs. Therefore, the CAM assay is a revolutionary technique that allows a simple and effective way to induce tumors, test the effectiveness of treatments, carry out metastasis studies, perform biopsy grafts of patients, and carry out personalized medicine. However, unification of the methodology used is necessary.

## 1. Introduction

Constant progress in understanding the mechanisms underlying tumor formation, as well as their migration and invasion into other tissues, is essential for the development of effective strategies in both cancer prevention and treatment. Traditional in vivo mice models have allowed us to obtain valuable information in this field, although they also present multiple limitations due to specific restrictions such as large cohorts of animals [1], slow tumor development, or ethical considerations [2]. In this context, egg models have emerged as promising tools that offer unique advantages not only in terms of accessibility, cost, and experimental manipulation [3] but also in terms of immune responses, as they are naturally immunodeficient hosts because until the late stages of incubation, the lymphoid system is not fully developed [4]. Because of that, in most countries, there is no need for ethical committees to approve this type of research if it ends on day 14 [5], making this another advantage of this type of model. 

During the development of the embryo within the egg, the mesodermal layer of the chorion, together with the allantois, fuses to generate the chorioallantoic membrane (CAM), thus connecting the embryonic circulation to the CAM, generating a large vascular network [4]. Since the CAM is not innervated, the embryo cannot feel pain [6]. The presence of the CAM provides nutrition for developing xenograft models due to its supportive environment surrounded by vessels that allows cell extravasation, ultimately leading to metastatic foci [3]. The CAM model can be used in cancer research as an alternative in vivo model to perform angiogenesis [7], tumorigenesis of solid tumors or cell suspensions [8], tumor chemosensitivity [9], and metastasis assays [10], among others. 

The two main egg models in tumorigenesis are the in ovo and ex ovo models, which are carried out inside and outside the egg, respectively. Both use the CAM for tumor development, although they have different advantages. The in ovo model allows for obtaining information on the extravasation of tumor cells and is mainly used for the study of metastasis [11], while the ex ovo model is mainly used in angiogenesis studies since it allows easier observation of the CAM [12].

Therefore, the aim of this systematic review is to search and discuss the literature over time on the use of the CAM model in cancer for tumorigenic assays in vitro or as a xenograft model and analyze the type of assay performed and the methodology used by the authors and relate it to the results obtained. 

## 2. Materials and Methods

This systematic review was previously registered in the OSF database on 10 November 2023 (https://doi.org/10.17605/OSF.IO/BN58M).

### 2.1. Study Eligibility and Data Sources

The present systematic review has been developed through a bibliographic search in four different databases: Cochrane, PubMed, SCOPUS, and Web of Science. To perform the search in PubMed, the following “MeSH” terms were used: “Chorioallantoic membrane” and “neoplasms”, with the formula obtained: “ovo”[All Fields] AND (“chorioallantoic membrane”[MeSH Terms] OR (“chorioallantoic”[All Fields] AND “membrane”[All Fields]) OR “chorioallantoic membrane”[All Fields]) AND (“analysis”[MeSH Subheading] OR “analysis”[All Fields] OR “assay”[All Fields] OR “biological assay”[MeSH Terms] OR (“biological”[All Fields] AND “assay”[All Fields]) OR “biological assay”[All Fields] OR “assays”[All Fields] OR “assayed”[All Fields] OR “assaying”[All Fields] OR “assays”[All Fields]) AND (“cancer s”[All Fields] OR “cancerated”[All Fields] OR “canceration”[All Fields] OR “cancerization”[All Fields] OR “cancerized”[All Fields] OR “cancerous”[All Fields] OR “neoplasms”[MeSH Terms] OR “neoplasms”[All Fields] OR “cancer”[All Fields] OR “cancers”[All Fields] OR “tumor” [All Fields]). In the case of the other databases, this formula was adopted. Moreover, this systematic review has followed the PRISMA guide to guarantee its correct execution [13].

### 2.2. Inclusion Criteria

Because in ovo tumor induction has been gaining great relevance in recent years, the literature search has not been restricted by publication date. Two types of studies were included: articles that treated these tumors and those that only generated tumors and studied their growth. Likewise, articles that have carried out studies on different species of birds have been included.

To reduce the possible risk of bias, after reviewing the bibliography of the articles included in the systematic review, those that met the inclusion and exclusion criteria were also added to this systematic review.

### 2.3. Exclusion Criteria

The main exclusion criteria were studies in which tumors were not induced, such as studies where they only used eggs to carry out angiogenic studies without tumor induction. Similarly, articles that did not specify the in ovo tumor induction methodology were excluded, although those that referenced previous works were included. Those whose methodology was incomplete but detailed the tumor induction process were also included. Articles that only used the egg to test biocompatible materials or scaffolds, without inducing tumors, were also excluded. Likewise, articles that were not research and were a protocol were also excluded.

Regarding language, articles that were written in a language other than Spanish, English, or French were excluded.

### 2.4. Study Selection 

G.P. and C.M. (Cristina Mesas) carried out the first bibliographic search independently and agreed on the search formula for each database, obtaining 357 articles. Once the articles were obtained, those that were not original articles, were not freely accessible, and were repeated, were excluded, obtaining a total of 273 articles.

**Figure 1:**
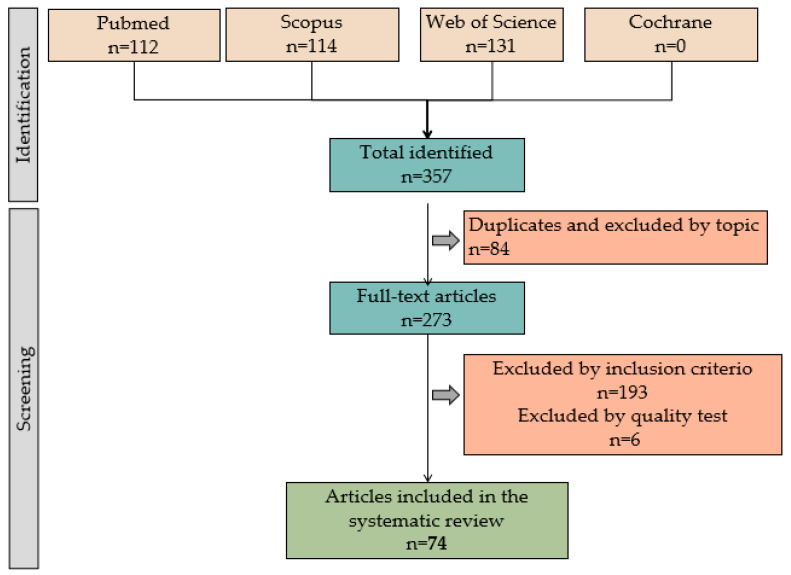
Flow diagram illustrating the search and selection process for articles included in this systematic review.

In the second step of the procedure, independently, M.A.C. and C.M. (Cristina Mesas) carried out a detailed reading of the articles, excluding those that did not meet the inclusion and exclusion criteria, obtaining a total of 74 articles, which were the ones that were finally analyzed in this systematic review (Figure 1).

### 2.5. Data Extraction

Following the procedure described, M.A.C. and C.M. (Cristina Mesas) carried out the procedure independently. According to Cohen’s kappa statistic test [14], there was a good correlation between M.A.C. and C.M. (Cristina Mesas) Any disagreements were resolved through discussion until a consensus was reached. Otherwise, a third experienced author made the final decision. Finally, each article was subjected to a quality test independently by M.A.C. and C.M. (Cristina Mesas). This quality test has two parts: the first consists of general filters on cell lines and patient biopsies (score ≥ 5). Articles that did not reach this score were excluded. The second phase consisted of questions about CAM methodology, results, discussion, and conclusions. The articles were classified according to the score obtained: low quality (score 0–5), medium quality (score 6–15), and high quality (score 16–20). Following this quality study, 6 articles were excluded (Figure 1), and 74 articles were included and are analyzed in Table 1, Table 2, Table 3, Table 4 and Table 5. The tables contain the references of the articles, the summarized CAM methodology, specifying the days on which the interventions on the eggs were carried out, and the techniques applied to study induced tumors. They also include the cell lines or patient biopsies used, as well as the main results obtained.

## 3. Results

**Figure 2:**
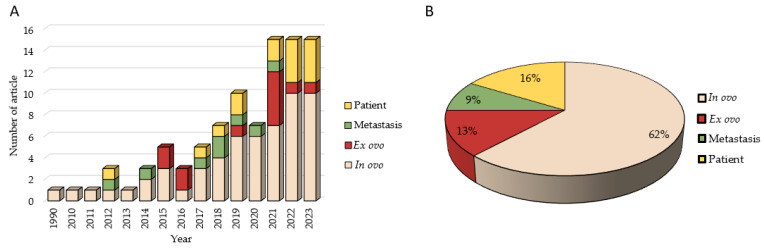
Graphic representation of (**A**) the number of scientific publications over the years considering the methodology used and (**B**) the methodologies used in tumor induction.

After the analysis described above, 74 articles have been analyzed in the systematic review (Figure 1). Tumor induction in the CAM has been gaining great interest in recent years. Figure 2A corroborates this with a graphical representation of the articles published per year using this technique, showing exponential growth. It is noteworthy that a substantial number of articles using this methodology began to be published only in 2018, reaching 15 annual publications within the last three years.

If we analyze the type of study that can be carried out on eggs to induce tumors, 62% of the articles induce tumors from cell lines using the in ovo technique, while 13% perform the ex ovo technique (Figure 2B). Likewise, it is worth noting that 16% of the studies carried out a xenograft methodology based on patient biopsies, with these studies being the most recent; they were conducted mainly in the last three years (Figure 2A). However, the study of in ovo metastasis is the least used technique (9%), although it has been performed in recent years (Figure 2B).

**Figure 3:**
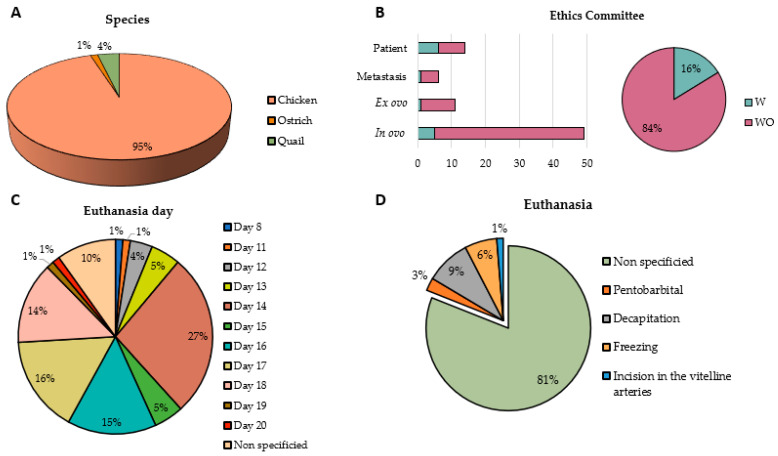
Graphic representation of (**A**) bird species used for in ovo experimentation, (**B**) articles that present the ethics committee’s approval for animal experimentation or not, (**C**) end point day on which embryo euthanasia is performed, and (**D**) methodology used to perform euthanasia on the embryo. W: with ethics committee approval; WO: without ethics committee approval.

After applying the inclusion and exclusion criteria, all articles were included regardless of the poultry species used to perform the CAM assay. As shown in Figure 3A, 95% of the articles used chicken as the bird species for the study. However, there is one article that uses ostrich eggs and three articles that use quail eggs.

Another key aspect of the use of fertilized eggs for experimentation is the ethics committee’s approval for animal experimentation. For fertilized eggs, according to different laws, they do not require ethics committee approval for their use in investigation. In fact, as seen in Figure 3B, 84% of the studies do not have this approval. Although 16% do have approval from the ethics committee, most articles specify that it is not necessary. The studies that use patient biopsies stand out as being the ones that provide the most approval from the ethics committee (Figure 3B).

There is a discrepancy between the end point of the experiment and, therefore, when the euthanasia of the embryo occurs. As can be seen in Figure 3C, most studies end the experimentation on day 14 (27%), followed by day 17 (16%), day 16 (15%), and day 18 (14%), highlighting that 10% of the articles do not specify the exact day of the end point. Furthermore, most articles do not specify how embryo euthanasia was carried out (81%). Among those that specify the procedure, 9% carry out euthanasia of the embryo by decapitation, 6% by freezing at −20 °C, 3% by intravenous injection of pentobarbital, and 1% by an incision in the vitelline artery (Figure 3D). 

### 3.1. Establishment and Tumor Formation in the CAM from Cell Lines following the In Ovo Methodology

**Figure 4:**
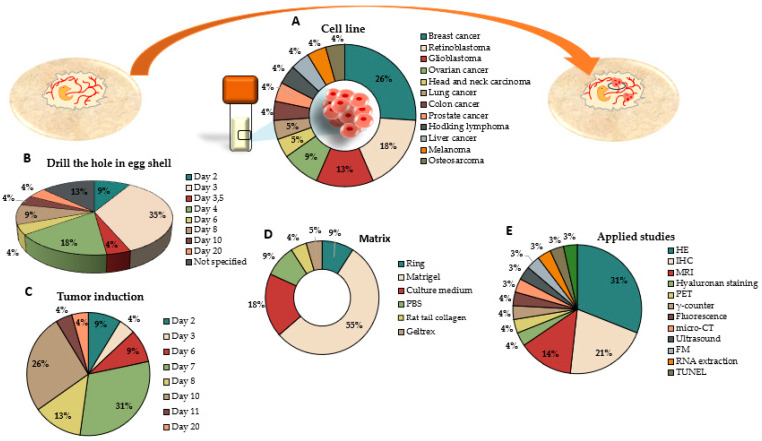
Graphic representation of the tumor induction procedure from cell lines in the CAM following the in ovo methodology, specifying (**A**) cell lines used to induce the tumor in the CAM, (**B**) the days in which the hole in the eggshell was drilled, (**C**) the day in which tumors were induced in the CAM, (**D**) the matrix used to induce the tumor, and (**E**) studies that were used in the study and characterization of induced tumors.

Among the 74 articles included in this systematic review, 48 induced tumors in the CAM from cell lines using the in ovo technique. Of these, 22 studied tumor genesis without treating them [15,16,17,18,19,20,21,22,23,24,25,26,27,28,29,30,31,32,33,34,35,36]. Successful results have been obtained in most studies, achieving high efficacy in tumor induction in the vast majority of tumor lines in which work has been carried out. There is great diversity in the types of cancer that have been tested for tumor induction in the CAM, with breast cancer cell lines being predominant (26%), followed by retinoblastoma (18%), glioblastoma (13%), and ovarian cancer (9%) (Figure 4A). 

A key aspect in carrying out this methodology is the day on which the hole in the eggshell is drilled. As can be seen in Figure 4B, there is much disparity in the day that it drills the hole. A total of 35% of the studies drilled the hole in the eggshell on day 3 of development, and 18% drilled it on day 4, highlighting that 13% of the studies did not specify the exact day. Similarly, this discrepancy was observed on the day of tumor induction, with 31% of studies inoculating tumor cells into the CAM on day 7 of development, followed by 26% on day 10 and 13% on day 8 (Figure 4C).

On one hand, the inoculation method for tumor cells is also not clearly established. In half of the studies analyzed (55%), tumor establishment was established with Matrigel, although culture medium (18%) and PBS (9%) were also used. However, other matrices are also used to establish tumor growth, as in the case of Gečys et al. (2023), who used rat tail collagen as the matrix for cell inoculation [16], or Yart et al. (2022), who used Geltrex [22]. On the other hand, it should be noted that only 9% of the studies used a silicone ring in the CAM to infiltrate the tumor cells into the CAM and facilitate its establishment at a fixed point, preventing its spread through the CAM (Figure 4D).

To assess the viability of the tumor induction assay and what this entails for the embryo, numerous techniques can be applied to provide information depending on the objective of the study. The most commonly used technique, in 31% of studies, was histology (Figure 4E), as in the case of Jaworski et al. (2013), who compared tumor formation in two different glioblastoma cell lines, where they observed histological differences in tumors formed between both lines using hematoxylin–eosin staining (HE) [34]. Another widely used technique is immunohistochemistry (IHC) in 21% of the cases (Figure 4E). Buschmann et al. (2022), performed immunohistochemical staining of HIF-1α to determine the hypoxic phenotype of tumors generated from lung cancer and colon cancer cells and also studied the positive values of the cell proliferation marker ki-67 [18]. 

In addition to conventional techniques for the study of tumors generated in vivo, complementary techniques, such as ultrasound, can also be used in the egg. Eckrich et al. (2020) obtained the tumor volume induced in the CAM from liver cancer cell lines by ultrasound and correlated it with other histological techniques, like hematoxylin and eosin (HE) stain [26].

Although the vast majority of studies were carried out in chicken embryos, there are experiences carried out in other types of embryos, as is the case of Gečys et al. (2023), who used ostrich embryos to carry out their studies on breast cancer, in which, based on known models of tumor generation in chicken embryos, they managed to establish the number of cells suitable for tumor induction in the ostrich CAM, and they also performed histological techniques that allowed them to determine the high proliferative activity of the tumor cells that they worked with [16] (Table 1).

Finally, studies using stem cells as a treatment for tumors, such as Gečys et al., who used adipose tissue-derived mesenchymal stem cell EVs [16], and Waltera et al., who used bone marrow-derived mesenchymal stromal cells [17], were analyzed. In both, stem cells were used to reduce tumor size and malignancy (Table 1). 

### 3.2. Tumor Induction Model in the CAM Using the In Ovo Technique to Determine the Effectiveness of Treatments

**Figure 5:**
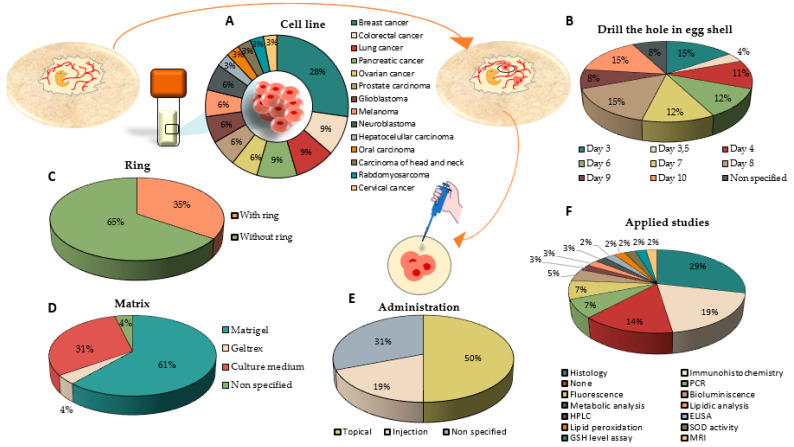
Graphical representation of tumor induction studies and in ovo treatment, highlighting (**A**) the type of cancer studied, (**B**) the day of embryonic development when the hole was drilled in the eggshell, (**C**) studies with and without the use of a ring, (**D**) studies that induce tumor formation by means of matrices or a culture medium, (**E**) route of administration of drugs, and (**F**) techniques applied in resected tumors.

Of the 48 articles that carried out in ovo tumor induction studies, 26 of them test different treatments on these induced tumors [37,38,39,40,41,42,43,44,45,46,47,48,49,50,51,52,53,54,55,56,57,58,59,60,61,62]. The most studied tumor types in this modality were breast cancer (28%), followed by colorectal, lung, and pancreatic cancer with 9% each, and prostate cancer, glioblastoma, melanoma, neuroblastoma, and ovarian cancer with 6% each. Finally, the least studied are cervical, hepatocellular, head and neck, and rhabdomyosarcoma cancer in 3% of the articles (Figure 5A). It should be noted that tumor formation was not successfully achieved in all the lines tested. Lung cancer cell lines SW1573, H1299, and H292 were not used to test the treatment because of the great irregularity of the tumors formed. In fact, only the A549 cell line formed solid tumors, while H460 formed tumors but was less compact [39].

The analysis of the methodology that followed the drilling of the hole in the eggshell was heterogeneous. In fact, the day of development on which the hole was opened varied among the studies. The preferred day by most studies was days 3, 8, and 10 (15% of studies in each case), followed by days 7 (12%), 6 (12%), 4 (11%), 9 (8%), and 3.5 (4%), and 8% of the articles did not specify the day on which the egg hole was opened (Figure 5B). Notably, in 15 of the 26 articles, tumor induction was performed on the same day in which the hole was opened [37,38,39,40,41,42,43,44,45,46,47,48,50,53,56,59].

On one hand, as shown in Figure 5C, 35% of the articles directly deposited the cells on the CAM. However, the majority of the articles (65%) deposited the CAM on a ring in which the cells were inoculated, acting as a barrier to prevent the spread of the cells through the CAM. On the other hand, many studies used cellular matrices containing the cells for tumor induction, such as Matrigel and Geltrex in 61% and 4% of the cases. In 31% of the studies, the cells were inoculated in a culture medium, and 4% of the published articles did not specify where the tumor cells were inoculated for the CAM (Figure 5D).

The in ovo tumors were developed for the purpose of testing treatments. Generally, one or two days after tumor induction, the tumors were treated. However, there are studies that induced tumors with previously treated cells, such as Mariho et al. (2018), who treated SKOV3 ovarian cancer cells with ApoA1 and CDDP, and later that day, deposited it in the CAM, continuing with the treatment to induce tumor formation [61]. Many studies employed chemotherapies such as Vincristine [37], cisplatin [38,39,43,49,50,53,61], 5-Fu [48], or Doxorubicin [48,56,59]. In addition, new therapies, such as plant extracts [51,55] or nanoparticles [38,49,58], have been tested. Treatments were administered directly on the tumor surface, that is, topically, in 50% of the cases (Figure 5E). However, there are studies, such as Waschkies et al. (2020), who used topical treatment on the tumor surface by means of air and carbogen flows directed to the tumor through plastic tubes [57]. Only 19% of the analyzed studies used injected treatments. While most of these treatments were injected into blood vessels, only one article performed intratumoral injections [59]. A total of 31% of them did not specify the route of drug administration (Figure 5E).

Once the tumor was resected, a wide variety of techniques were used for its analysis (Figure 5F). The most commonly applied technique was histology by HE staining (29%), followed by immunohistochemistry (19%), PCR and fluorescence (7%), and bioluminescence (5%). Other techniques were used to a lesser extent, such as metabolic and lipid analysis, HPLC, ELISA, lipid peroxidation assays, SOD activity, GSH levels, and MRI. A total of 14% of the articles did not employ any alternative technique for tumor study other than microscopy imaging (Figure 5F).

In all included studies, the treatments were effective, and a significant decrease in the tumor volume induced in the CAM could be observed with respect to the untreated controls. In fact, it was also possible to corroborate, in the case of the study carried out by Bohm et al. (2019), that treated tumors also showed a greater capacity for CAM invasion, a higher degree of vascularization, and a more aggressive phenotype [62] (Table 2).

### 3.3. Effectiveness of Tumor Induction following the Ex Ovo Methodology

**Figure 6:**
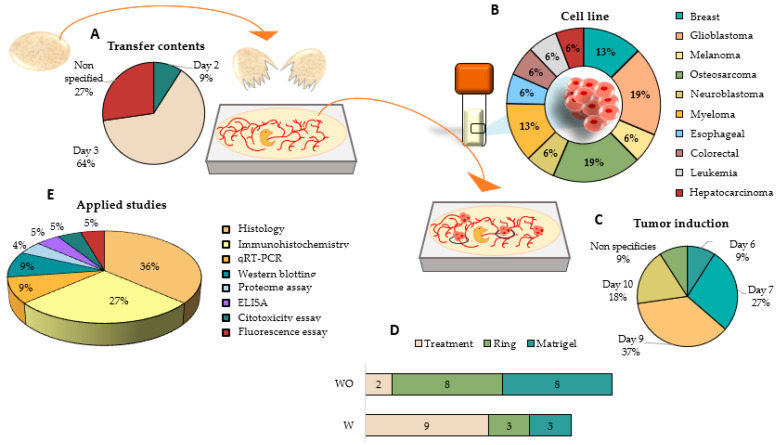
Graphic representation of tumor induction in the CAM following the ex ovo methodology including (**A**) the day that they break the egg and transfer its contents to a plate, (**B**) cell lines used to induce the tumor, (**C**) the day on which tumors are induced in the CAM, (**D**) information on treatment received and tumor induction methodology, and (**E**) studies used to study the tumors generated. W: with ethics committee approval; WO: without ethics committee approval.

Eleven articles included in this systematic review [38,53,63,64,65,66,67,68,69,70,71] carried out tumor induction in the CAM using the ex ovo model by transferring the contents of the egg to a plate. The most common day on which studies break the egg and transfer its contents to a plate is day 3 (64%), followed by day 2 (9%), although 27% of the studies do not specify the day (Figure 6A).

The most commonly used cancer cell lines in ex ovo experimentation in this model were osteosarcoma and glioblastoma (both at 19%), followed by breast cancer and myeloma (both at 13%) (Figure 6B). 

There is greater variability in terms of the day of tumor induction, including day 9 of development in 37% of the cases, followed by day 7 (27%) and day 10 (18%) (Figure 6C).

The most commonly used method for induction was direct treatment by injection, although there is the possibility of using a silicone ring or Matrigel for localized induction of the tumor (Figure 6D).

Treatment of tumor cells is usually performed on the same day or after tumor cell implantation, although there are two articles in which the cells implanted in the CAM have been previously treated with the drug [53,64]. In the study by Merlos et al. (2021), the treatment of the cells did not refer to the use of a drug, but cell culture was performed beforehand in the presence or absence of Matrigel to evaluate the difference in tumor growth and development in both cases [53]. 

Most assays performed to study tumor formation were histology and immunohistochemistry with 36% and 27%, respectively, although qRT-PCR, Western blotting, proteasomal assays, ELISA, cytotoxicity, and fluorescence assays had also been performed (Figure 6E). 

All articles that used the ex ovo methodology obtained favorable results, both in correct tumor induction and in its reduction after treatment. There are studies in which they treat the cells before inducing the tumor [64,66], while others perform the treatment once the tumor is established in the CAM [38,53,63,69].

### 3.4. Use of the CAM Assay as a Model for Metastatic Induction and to Study the Effectiveness of Treatments

**Figure 7:**
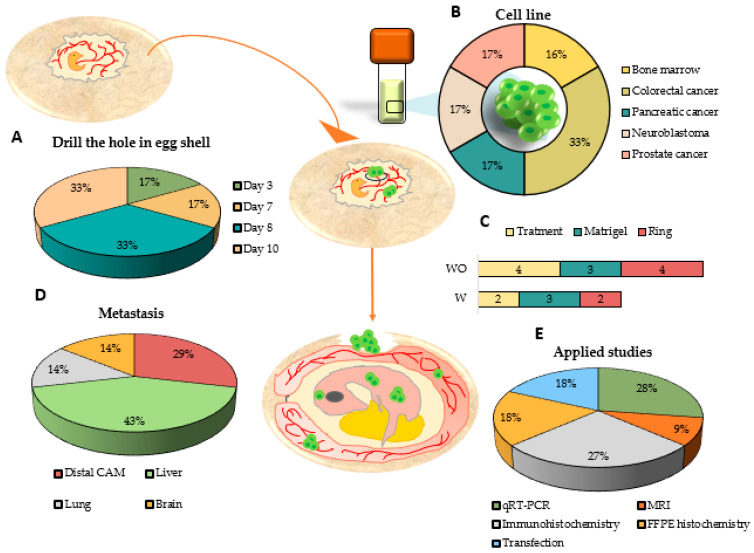
Graphic representation of the use of the CAM assay for the study of in ovo metastasis formation, analyzing (**A**) the day in which the hole in the eggshell was drilled, (**B**) the cell lines used to induce the tumor, (**C**) the methodology carried out to induce the tumor in the CAM, (**D**) the main organs where metastases have been observed, and (**E**) the applied studies that can be used to characterize metastasis or tumor induction. W: with ethics committee approval; WO: without ethics committee approval.

In ovo studies have been used as metastatic models, verifying their effectiveness in the formation of metastases in different organs of the embryo; they have also been used to test different treatments and verify their effectiveness in tumor migration. In this systematic review, six articles used the CAM assay to induce metastasis formation in ovo [53,62,72,73,74,75]. In these studies, the main day on which the hole in the eggshell is opened was days 8 and 10 (33% each one), followed by days 3 and 7 (17% each one) (Figure 7A). Colorectal cancer is the most used cell line for study in ovo metastasis (33%), although pancreatic cancer, neuroblastoma, prostate cancer, and bone marrow have been tested (Figure 7B). During tumor induction, only two articles used a silicon ring, while three articles embedded the tumor in Matrigel to ameliorate tumor induction. In addition, to study the formation of metastasis both in the CAM and the embryo, studies have been carried out on the effectiveness of treatments in reducing metastasis in two articles (Figure 7C). An example of this is Pawlikowska et al., who carried out assays with non-small-cell lung cancer and prostate cancer tumor cell lines. They performed metastasis assays in several lines of both tumors, which were analyzed using macroscopic fluorescence and 3D imaging studies. In addition, they tested chemotherapy drugs, like cisplatin and docetaxel, and their effect on metastasis. The results obtained showed a decrease in metastasis of the CAM and the chick embryo after pharmacological treatment [75]. Merlos et al. carried out an in ovo study to determine the metastatic capacity of two antitumor drugs, cisplatin and ellipticine. They used the UKF-NB-4, a human neuroblastoma tumor cell line to induce tumors in fertilized chicken eggs. Six days after induction, they performed a qPCR with human-specific Alu sequence primers, both in the CAM and in different organs such as the liver, brain, and lungs. In their results, they obtained a significant reduction in the extravasation of cancer cells to the distal organs, although the authors consider that this may be due to the antitumor effect of the drug itself and not a specific antimetastatic effect [53]. Although only two articles test direct treatments in ovo, there are also articles that perform prior treatments on cell lines before inducing the formation of metastasis. This is the case of a study that used Alu sequences to demonstrate that by inhibiting miR-21, the metastatic capacity of tumor cells from the LS174T colon cancer cell line is statistically decreased in an in ovo model. To perform this, they transfected the tumor cells with LNA-anti-miR-21, thus silencing the expression of this miRNA. Metastasis was not reduced in those groups in which miR-21 was not silenced, which could be a future target in tumor treatment [74] (Table 4).

The effectiveness of the trial has been demonstrated through the analysis of metastases, with the organs with the most prevalence in the formation of metastases being the liver (43%), followed by the distal CAM in 29% of the studies and the lungs and brain (14% each) (Figure 7D).

Metastases produced in eggs can be detected by different methodologies, such as qRT-PCR (28%) and immunohistochemistry (27%) (Figure 7E). Herrman et al. carried out different types of in ovo studies, concluding that MRI was capable of being a sensitive technique for detecting metastasis deposits of at least 12 cells. They performed this with two approaches, one of them by injecting hypoxic fluorescent tumor cells SK-N-AS (GFP SK-N-AS) directly into the brain of white leghorn chicken embryos and the second one by inducing a tumor in the CAM through SK-N-AS fluorescent cells loaded with micron-sized iron particles (GFP MPIO-labeled SK-N-AS) [73]. 

### 3.5. Efficacy of the Patient-Derived Xenograft Model and Its Use to Predict Chemotherapeutic Drug Sensitivity/Resistance

**Figure 8:**
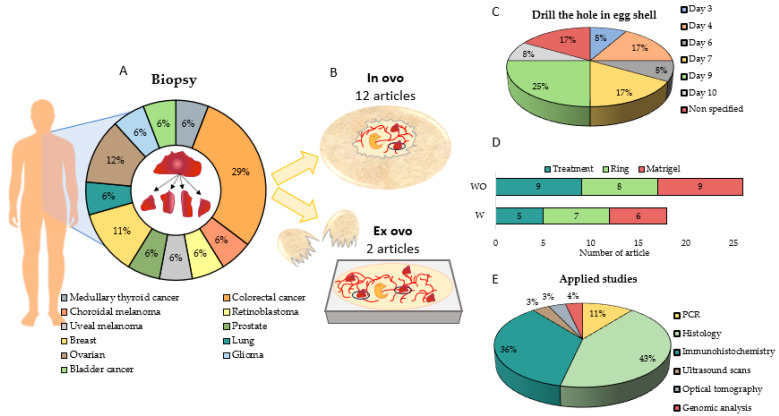
Graphic representation of the procedure to induce CAM tumors from patient biopsies, analyzing (**A**) the type of tumor of origin from which the biopsy is obtained, (**B**) the type of methodology used in the CAM test, (**C**) the day on which the hole in the eggshell, in the case of the in ovo method, is drilled, (**D**) the number of articles that treated the tumor and used a ring and Matrigel to ameliorate the induction, and (**E**) the main studies that can be applied to analyze the formed tumor. W: with ethics committee approval; WO: without ethics committee approval.

In the last 3 years, publications using the CAM assay for tumor induction from patient biopsies have increased significantly (Figure 2A). Of the 14 articles included in the systematic review about this method [1,71,76,77,78,79,80,81,82,83,84,85,86,87], 29% used a biopsy from colorectal cancer, followed by ovarian cancer (12%) and breast cancer (11%) (Figure 8A). Regarding methodologies, only two articles followed ex ovo methodology [71,83], while the rest of the articles used in ovo assays (Figure 8B). In the rest of the results, there has been also a discrepancy regarding the opening day of the hole in the eggshell, and day 9 was the day in which the majority drill the hole (25%), followed by days 4 and 7 (17% in both). It should be noted that 17% of the articles do not specify the exact day (Figure 8C). 

Regarding tumor induction, seven articles used a silicon ring in the CAM during tumor induction, and only six articles mixed a fragment of biopsy with Matrigel before induction in the CAM (Figure 8D). Tsimpaki et al. (2023) used the CAM assay as a xenograft model of biopsies from patients with uveal melanoma, analyzing different implantation methodologies. For this, different methodologies were carried out: (i) direct implantation, (ii) implantation on a drop of Matrigel previously placed in the CAM on a lacerated blood vessel, or (iii) implantation on a drop of Matrigel placed in the center of the ring. Tumors were successfully induced in all groups, although there were differences in tumor dissemination, depending on whether they were in the ring or not. In addition, they carried out numerous studies (ultrasound, scans, fluorescein angiography) that allowed them to obtain deeper analyses of tumor formation [78]. Although detailed studies can be carried out, 43% of the analyzed studies performed histology analysis, and 36% of them carried out immunohistochemistry studies (Figure 8E).

The results in all articles showed good implantation of the biopsy fragments; in addition, when treated with the chemotherapy used in the clinic, a predictive response correlation was observed between the egg and the patient’s response. Charbonneau et al. (2023) conducted a study in which they biopsied 60 patients with glioma who were being treated with CB and TMZ. Biopsy fragments were implanted into the CAM, and treatments were injected IV into the CAM vasculature. After analyzing the results obtained, 98.3% of the biopsies were successfully established in the CAM, in addition to maintaining the histopathological and molecular characteristics of the original tumor. Additionally, there was a correlation between the patient’s response to chemotherapy and the in ovo response [71]. 

Another aspect to highlight is that primary tumors grafted onto the CAM as ground homogenates adopted a different morphological phenotype compared to the original tumors, while fresh tumors grow well and show similar histology to the original tumor. Moreover, ki-67 expression was better retained in tumors grafted from frozen samples compared to fresh ones [86].

## 4. Discussion

For hundreds of years, the use of fertilized chicken eggs has been crucial for the investigation of embryonic development. Thanks to these studies, very important concepts, such as the neural tube or the germ layers, have been described [88]. Currently, the study of the chicken egg and, in particular, its CAM, is gaining great importance in various fields. In biomedicine, as discussed in this systematic review, its application in tumor induction studies and testing of antitumor treatments is widespread [37]. In addition, many studies focus on the search for antiangiogenic drugs using the CAM vascular network [89]. Recently, the CAM has started to be used in tissue engineering for organoid implantation. Moeinvaziri et al. (2021) implanted an otic organoid generated from pluripotent stem cells, mesenchymal stem cells, and endothelial cells into the CAM. Its implantation in the CAM stimulated the maturation of cells similar to human hair cells [90]. Within regenerative biomedicine, the CAM can be used as a bioreactor to culture and study human living bone regeneration [91]. It is even very useful for the biological characterization of materials through irritability testing. Chen et al., 2018, implanted bioactive collagen–bioglass scaffolds and did not detect inflammation or necrosis in the membrane, demonstrating its biological compatibility [92]. On the other hand, since the use of the chick embryo has the advantage of developing outside the mother, there has been great interest in its application for studies in the field of epigenetics [93]. Of particular note is the application of the CAM in the field of microbiology, where the virulence, invasiveness, and pathogenicity of bacteria and yeasts are studied [94].

Nowadays, the CAM assay has become increasingly relevant for the study of various cancer processes, including tumor induction. This assay has several advantages over mouse experimentation, one of them being the high vascularization and immature immune system, which allows experimentation with cell lines or tissues from different species without immune response [88,95]. In addition, the chorioallantoic membrane model is considered an in vivo model that fits the 3R principle of animal experimentation, replacement, reduction, and refinement [96]. Another advantage of this model over the use of mice for experimentation is that no ethical approval is required for its use, as chick embryos do not develop the nervous system until day 17 of ontogenesis [97], so experiments are terminated before the development of the regions associated with pain. However, The National Institute of Health and the Institutional Animal Care and Use Committee (IACUC) [98] state that chick embryos can be used without any ethical restrictions until day 14 of gestation, as they lack pain perception. There are studies that confirm that pain sensation in chicken embryos is impossible up to incubation day 7, but there are no specific time points defined from which the chicken embryo is able to develop the nociception and pain sensation [99]. In our systematic review, there is a disparity in the days on which researchers euthanize chick embryos, although in 27% of studies, euthanasia occurs on day 14, followed by 16% of the studies in which it occurs on day 17 (Figure 3C). This disparity may be due to the lack of a clear protocol regarding the end point of the methodology. Most authors use day 14 or earlier since approval by the ethics committee is not required until day 14, although other factors, such as the type of sample used in the study and the type of study performed (in vivo or ex vivo), also affect these differences. However, it would be desirable to unify criteria and establish a single end point day for chick embryo research.

The results analyzed from the articles included in the present systematic review show the effectiveness of inducing tumors in the egg CAM from different tumor types. Tumors have been successfully induced from in vitro cell lines of different tumor types, and the most studied are breast cancer, colorectal cancer, lung cancer, glioblastoma, and pancreatic cancer cell lines. These lines have been used in both in ovo and ex ovo, which are the types of cancer with the highest incidence and mortality all over the world [100]. In metastasis formation studies, pancreatic cancer lines are the most used, which is understandable since this tumor, despite not having a high prevalence, presents a high mortality rate [101]. For tumor induction from patient biopsies, the most studied tumors are those whose biopsies are easy to acquire, such as colorectal cancer, retinoblastoma, and ovarian cancer. Seeing the effectiveness of tumor induction on the egg CAM, studies should be carried out with other cell lines, such as liver or stomach cancer, which also have a high prevalence and mortality in the population.

There is a large discrepancy in the day on which the hole is opened in the eggshell. It is generally opened on day 3, although in studies of metastasis formation or tumor induction from patient biopsies, it was opened on day 9 or 10. Perhaps this discrepancy is due to the fact that in the case of tumor formation from cell lines, 6 days are necessary to consolidate tumor formation, while in the case of patient biopsies, these are already consolidated and can be implanted in later days of embryonic development. The same would occur in the case of metastatic studies where the cells are injected IV. Specific studies would be necessary to report on the risks of opening the window in the eggshell on different days. It should be noted that although tumor induction was effective without any matrix, the use of Matrigel is used among all studies in all types of assays. The extracellular matrix (ECM) forms the non-cellular physical support for the cellular constituents of all tissues and organs. The components of the ECM encompass cellular and biomechanical signals that maintain morphogenesis, differentiation, tissue homeostasis, integrity, and elasticity [102]. The use of Matrigel promotes correct tumor formation since it provides an ideal tumor microenvironment for its growth and differentiation [103,104].

Nowadays, there are studies aimed at demonstrating the potential of the CAM as a study model for precision medicine. Currently, mice are used for such studies, which has some drawbacks, such as the time required, the high cost, or the requirement for approval of the experiment by the ethics committee [37]. Also, the use of the CAM allows direct observation of the evolution of the tumor mass. It has been observed that tumor cells engrafted in the CAM behave similarly to the patient’s tumors, such as angiogenesis, metastasis, or matrix interaction. In addition, the implantation of tumor biopsies allows the recovery of some characteristics of the primary tumor, such as cellular polymorphism [97]. For the drug effect, these appear to behave the same in mice and the CAM, but the sub-toxic dose limit is lower in the chicken model, causing the death of the embryo [48]. On the other hand, the dose supported by the mouse is higher, and the toxicity in this model is reflected by a reduction in weight. In addition to the reduced toxicity threshold, the CAM study has certain limitations that are overcome by the use of humanized mice, such as the use of genetic modifications of HLA or cytokines [97]. Even so, the CAM assay allows a first study to estimate the sub-toxic dose that can be used in the mouse and make an approximation of the effect of the drug, avoiding the need for a first experiment with the mice [48].

The limitations found while developing this systematic review were focused on the lack of information in the articles’ methodologies. Many of them did not present complete methodological information regarding the day of egg opening, tumor induction, or end point day, which are very relevant pieces of information when it comes to standardizing processes. This causes a lot of variability to occur between the analyzed studies. Likewise, a gap has been found regarding the method used for euthanasia of the embryos. Although approval by the ethics committee of these processes is not necessary as long as they are carried out before the 14th, some rules should be established for their completion, such as specifying the end point day and the euthanasia method.

## 5. Conclusions

In this systematic review, the utility of the chicken egg CAM assay method in biomedical research has been comprehensively explored, with a specific focus on tumor induction and the evaluation of antitumor treatments. Over the years, the use of the CAM has evolved from its fundamental role in the study of embryonic development to become a valuable tool for investigating a wide range of biological and medical processes. The results reveal the effectiveness of the CAM model in inducing tumors from various cell lines, from high-incidence cancers, such as breast and colorectal, to less common types. The versatility of the model has allowed not only the successful induction of tumors in ovo and ex ovo but also the evaluation of antitumor treatments with promising results. This review highlights the importance of unifying criteria in the methodology, such as the day of egg opening, the method of tumor induction, and the end point of the experiment. Furthermore, the relevance of the extracellular matrix, especially the use of Matrigel, in the adequate formation of tumors is highlighted, providing an ideal microenvironment for tumor growth and differentiation. The CAM is presented as a valuable alternative to traditional mouse models, offering important advantages, such as high vascularization, an immature immune system, and the absence of the need for ethical approval for studies up to day 14 of gestation. Furthermore, its application in precision medicine seems promising, providing the opportunity to directly observe the evolution of tumor masses and recover characteristics of primary tumors through the implantation of biopsies. Despite the important advances and contributions of the CAM model, this review highlights the need for greater standardization and transparency in the presentation of methodological data in the scientific literature. The lack of detailed information in some studies analyzed represents a challenge in the comparison and synthesis of results. This systematic review highlights the crucial role of the CAM in biomedical research, particularly in the field of oncology. Opportunities to improve methodological coherence are identified, and the importance of continuing to explore the potential of this model in various areas of scientific research is highlighted.

## Figures and Tables

**Table 1:** The CAM assay as a methodology for tumor induction in ovo from cell lines.

Ref.	Methodology	Cell Line	Applied Techniques	Results
[15]	Day 20: window and tumor induction Day 34: end point by pentobarbital	Breast cancer: MDA-MB-231: − 1 × 10^6^ cells/egg in Matrigel 70% in 2 eggs − 2 × 10^6^ cells/egg in serum-free culture medium and Matrigel in 5 eggs − 6 × 10^6^ cells/egg in Matrigel 60% in 1 egg	HE	No tumor growth was observed in the CAM of the ostrich embryo when 1 × 10^6^ cells were inoculated. Fourteen days after inoculation of 2 × 10^6^ cells, tumor growth was observed in two of the ostrich embryos. Inoculation of 6 × 10^6^ tumor cells also did not result in successful tumor formation. Histological analysis showed a diffuse proliferation of epithelial tumor cells within the mesenchymal stroma of the CAM. The tumor cells showed high proliferative activity with numerous mitotic cells.
[16]	Day 3: DESH and tumor induction Day 7: sponge implantation Day 12: end point	Glioblastoma: − HRO636, U87, MG, and T98G: 1 × 10^6^ cells/egg in rat tail collagen type I. Treated with 5 μg/mL of ASC-EV for 48 h ASC-EV	HE	Untreated tumors of all cell lines showed diffuse growth. Tumors treated with ASC-EV showed sharper contours and growth on the CAM surface without deep penetration. HE revealed in the control group tumor cell invasion into the mesenchyme of the CAM, ASC-EV treatment reduced tumor invasion of the CAM, and the CAM of treated tumors showed less thickening and fewer blood vessels.
[17]	Day 3: DESH Day 7: tumor induction Day 14: end point	BMSCs Squamous cell carcinoma of the head and neck: − SCC9 Both were used in monoculture or co-culture. Inoculation in monoculture and co-culture included 2 × 10^6^ cells/egg in Matrigel Cells were pre-treated with 10 µM of the MMP-9-specific inhibitor JNJ0966 on day 1 and day 3	-	Eggs whose cells were treated with specific MMP-9 inhibitors showed a significant reduction in tumor size compared to the controls.
[18]	Day 3, 5: DESH Day 7: tumor induction Day 14: end point	Lung cancer: − A549, H460: 0.5 × 10^6^ cells/egg in growth factor-reduced Matrigel Murine colon cancer: − MC-38: 0.5 × 10^6^ cells/egg in growth factor-reduced Matrigel	MRI, IHC	A549 tumors showed a significant T1 response during HCHO. H460 or MC-38 tumors did not respond to HCHO. HIF-1α immunohistochemical staining corroborated the hypoxic phenotype of H460 and MC-38. In addition, ki-67-positive cells were found for H460.
[19]	Day 8 and 10: DESH and tumor induction Day 17: end point by decapitation.	Retinoblastoma: − Y79, WERI-Rb1, and RB355 All of them were inoculated at different concentrations: 1 × 10^6^; 2 × 10^6^; and 3 × 10^6^ cells/egg in PBS	-	All cell lines showed less tumor formation after inoculation at day 8 compared to inoculation at day 10. Inoculation of different cell concentrations did not change tumor size and weight. Inoculation of 1 × 10^6^ cells is adequate and sufficient for inoculation on day 10, leading to an average size of 6 mm and weight of 34 mg.
[20]	Day 3: DESH Day 10: ring and tumor induction Day 16: end point	Breast cancer: − MDA-MB-361 and Bt-474: 10^6^ cells/egg in 50% Matrigel Both were transfected with either control siRNA or SORL1	-	SorLA silencing inhibited tumor growth in both cell lines.
[21]	Day 4: DESH Day 8: tumor induction Day 13: end point	Breast cancer: − MCF10CA and MCF10A: 10^6^ cells/egg in PBS and Matrigel (1:1)	IHC, hyaluronan staining	MCF10CA cells showed higher proliferation and formed larger tumors than MCF10A cells. Tumor cell-associated hyaluronan was high in MCF10CA, while MCF10A cells had less intense staining. For ki-67 staining, more positive cells were observed in the MCF10CA line.
[22]	Day 8: DESH and tumor induction Day of end point not specific	Ovarian cancer: − SKOV3-red: SKOV3 transfected with the Ph2b-mCherry-IRES-puro2 plasmid selected with puromycin − SKOV3-M-green: SKOV3 transfected with the pEGFP-CenpA-IRES-neo plasmid selected with G418 − SKOV3-M:SKOV-red and SKOV3-green were co-cultured (1:1) and puromycin and G418 were added From all of them, 2 × 10^6^ cells/egg in Geltrex	-	No significant differences in tumor size and growth were found in the SKOV3-M line compared to the parents (SKOV—red and SKOV—green).
[23]	Day 2: DESH Day 5: place silicone ring Day 6: tumor induction Day 13 and 15: treatment Day of end point not specified	Prostate carcinoma: − PC-3 (PSMA−): 1 × 10^6^ cells/egg in 30% Matrigel − LNCaP C4-2 (PSMA+): 2 × 10^6^ cells/egg in 30% Matrigel	MRI PET HE γ-counter IHC	LNCaP C4-2 reached a volume of 0.025 ± 0.008 mL and PC-3 0.023 ± 0.011 mL for PC-3 after 8 days of growth. HE showed clearly visible tumors in the CAM. PSMA labeling was detected in C4-2 LNCaP tumors, while PC-3 tumors were negative.
[24]	Day 3: DESH Day 7: tumor induction Day of end point not specified	Breast cancer: − MDA-MB-231, shPHGDH-C3, and shPHGDH-D4 (transfected lines obtained by knocking down PHGDH using two different shRNAs): 2 × 10^6^ cells/egg in Matrigel	Fluorescence	ShPHGDH-C3 and shPHGDH-D4 tumor volumes were smaller than the controls at 24% and 39%, respectively.
[25]	Day 3: DESH Day 10: tumor induction Day 14: end point	Hodgkin lymphoma: − L428 and L1236: 2 × 10^6^ cells/egg in Matrigel Combination with macrophages: 1 × 10^6^ cells in Matrigel	Micro-CT, IHC	The micro-CT assay showed that LM was smaller and without hemorrhages compared to LWM. CD30 revealed that LWM invaded the entire CAM, while LM was compartmentalized in the upper and lower part of the tumor and was absent in the center. Prox1 revealed the absence of lymphatic vessels, while LM presented lymphatic vessels in the area of invasion of the CAM, favoring the diffusion of lymphoma cells.
[26]	Day 3: DESH Day 7: tumor induction Day 14: end point by decapitation	Liver cancer: − HuH7: 5 × 10^6^ cells/egg in Matrigel	Ultrasound, HE	A total of 50.39% of the eggs maintained viability and formed tumors. The tumor volume obtained by ultrasound was 0.69 cm^2^, and by HE 0.096 cm^2^, they were significantly correlated. The tumor vascularization obtained by histology and ultrasound was correlated.
[27]	Hole opening day not specified Day 10: tumor induction Day 17: end point	Retinoblastoma: − Y79 The number of cells and where they were suspended is not specified	_	Tumors from EMP1-overexpressing cells exhibited greater size, weight, and volume than the controls that did not overexpress EMP1.
[28]	Hole opening day not specified Day 2: tumor induction Days 10–17: end point	Retinoblastoma: − Y79RB: 50 µL of Y79RB supernatant overexpressing *TFF1*	FM, RNA extraction	Stable, lentiviral *TFF1* overexpression reduces tumor formation capacity of Y79 and RB355 cells in CAM assay results.
[29]	Day 4: DESH Day 10: tumor induction Day 18: end point	Melanoma: − A375: 10^4^ cells/egg in culture medium	-	Tumor formation was successfully induced from the A375 cell line on the CAM; tumors were compact on day 4 and had sizes of 2.2 ± 0.4 mm^2^ and 1.5 ± 0.3 mm^2^, respectively. Furthermore, great angiogenesis was observed around the formed tumors.
[30]	Day 4: DESH Day 7: tumor induction Day of end point not specified	Breast cancer: − MDA-MB-231: 2 × 10^6^ cells/egg in 50% Matrigel	MRI	In ovo MRI can be used for assessment of the in vivo biodistribution of labeled compounds, thus enabling efficient non-invasive initial testing.
[31]	Day 2: DESH and tumor induction Day 10–17: end point	Retinoblastoma: − Y-79 RB: 1 × 10^6^ cells/egg *TFF3* overexpressing, GFP-labelled or control cells in PBS. A total of 1.5 × 10^5^ cells/egg TFF3 overexpressing or control cells in culture medium	HE	The CAM assays revealed that *TFF3* overexpression influences anchorage-independent growth and significantly decreases the size of tumors forming from retinoblastoma cells.
[32]	Day 4: DESH Day 7: tumor induction Day 16: end point	Breast cancer: − MDA-MB-231: 2 × 10^6^ cells/egg in 50% Matrigel	MRI	High-resolution magnetic resonance imaging can be used as an effective technique to monitor tumor growth in ovo.
[33]	Day 6: DESH and tumor induction Day 18: end point	Glioblastoma: − U87-MG: 5 × 10^6^ cells/egg in culture medium	HE, IHC, TUNEL	Tumor induction in ovo allows the tumor tissue to maintain the biological characteristics corresponding to primary glioblastoma multiforme.
[34]	Hole opening day not specified Day 7: tumor induction Day 17: end point	Glioblastoma: − U87, U118: 3–4 × 10^6^ cells/egg in culture medium	HE, TEM	Tumors were successfully induced in the CAM of the egg from glioblastoma cell lines (U87 and U118).
[35]	Day 3: DESH Day 11: tumor induction Day 14: end point	Ovarian cancer: − OVCAR-3: 9 × 10^5^ cells/egg in Matrigel	HE, IHC	The CAM assay is a robust and cost-effective model for the testing of new bioactive antitumor agents, as it is an effective model for the study of ovarian cancer cell metastasis.
[36]	Day 3: DESH Day 10: tumor induction Day 17: end point	Different osteosarcoma cell lines at different concentrations.	HE	The CAM assay allows tumor development from osteosarcoma cell lines, making it possible to use it for the preclinical detection of anticancer molecules.

Alpha-SMA (alpha smooth muscle actin); ASC-EVs (adipose tissue-derived mesenchymal stem cell EVs); BMSCs (bone marrow-derived mesenchymal stromal cells); DHES (drill a hole in the eggshell); EMP1 (epithelial membrane protein 1); FM (fluorescence microscopy); HCHO (hypercapnic–hyperoxic); HE (hematoxylin and eosin); IHC (immunohistochemical); LMs (lymphomas with macrophages); LWMs (lymphomas without macrophages); micro-CT (microcomputed tomography); MMP-9 (matrix metalloproteases 9); MRI (magnetic resonance imaging); PBMCs (peripheral blood mononuclear cells); PSMA (prostate-specific membrane antigen); PHGDH (phosphoglycerate dehydrogenase); SorLA (sortilin-related receptor); TEM (transmission electron microscopy); TFF1 (trefoil factor 1); TFF3 (trefoil factor 3).

**Table 2:** CAM assay as a methodology used to evaluate the efficacy of antitumor treatments on tumors induced from cell lines.

Ref.	Methodology	Cell Line	Treatment and Administration	Applied Techniques	Results
[37]	Day 9: DHES and silicone ring and tumor induction Day 12: treatment Days 14, 16, and 18: end point by overdose of pentobarbital	Rhabdomyosarcoma: − RD and SJ-Rh30: 2 × 106 cells in Matrigel and PBS (3:1)	VCR at 1 nM, 10 nM, and 1 μM Administration: topical	HE IHC	Tumor cells were positive for human-specific vimentin, as confirmed by IHC. Antibodies against human vimentin did not cross-react with chicken tissues. The volume of resected tumors decreased in a concentration-dependent manner. In addition, the necrotic spread was concentration-dependent.
[38]	Day 10: DHES and tumor induction Day 16: treatment End point day: not specified	Breast cancer: − MDA-MB-231: 1.5 × 10^6^ cells in medium	CDDP at 100 μg/mL PtNPs-10 and PtNPs-40 at 250 μg/mL Administration: topical	HE FC	PtNPs-10 achieved greater tumor growth inhibition, while growth inhibition by CDDP was not significant. HE staining showed that cells migrate from the primary tumor, invading the nearby CAM. Tumors treated with CDDP and PtNP showed partial disintegration of the primary tumor. PtNPs-40 induced a visible degradation of the tumor into smaller fragments scattered around the primary tumor.
[39]	Day 6: DHES and tumor induction Days 9 and 11: treatment Day 18: end point by freezing at −20 °C overnight	NSCLC: − SW1573, A549, H1299, H292, and H460: 1 × 10^6^ cells in Matrigel	Pemetrexed at 1.5 to 20 mg/kg CDDP at 0.1 to 3 mg/kg Pemetrexed at 10 mg/kg with CDDP at 10 mg/kg Administration: not specified	HE IHC bioluminescence	Because of their irregularity in forming tumors, lines SW1573, H1299, and H292 were not chosen for treatment. Line A549 formed solid tumors, while H460 formed less compact tumors. The percentage of Ki-67- and APE1-positive cells in H460 tumors was approximately 100%, while in A549, the majority of cells were Ki-67-negative Combination chemotherapy decreased the tumor in A549, while the size of the treated H460 tumors was not evaluated, possibly due to extensive tumor cell synthesis
[40]	Day 8: DHES and tumor induction with spheroid Day 12: treatment Day 14: end point by freezing at −20 °C	Breast cancer: − BT-474, SK-BR-3, MCF-7, and MDA-MB-231: spheroids (5000 cells) in Matrigel	ALA and PSI-ALA-Hex 33 at 100 and 300 μmol/kg Administration: intravenous	FC	ALA induced the highest PpIX selectivity at 300 μmol/kg. After day 4 of injection, all spheroids reached maximal selectivity, except MCF-7.ALA, which is able to induce PpIX accumulation in all breast spheroids. PSI-ALA-Hex induced the highest selectivity in all lines at 300 μmol/kg, although it was lower than ALA.
[41]	Day 7: DHES, silicone ring, and tumor induction Day 14: treatment Day 17: end point	Breast cancer: − -MDA-MB-231: 3 × 10^6^ cells in medium	M at 10 µg/mL GN and GO, MGN and MGO at 20 µg/mL Administration: injection	ELISA lipid peroxidation assay, SOD activity, and GSH-level assay	No significant differences in tumor mass and volume were observed. Significant increase in caspase 3 and 8 in all treatments, with the greatest increase observed with M and MGO treatments. Increase in MDA concentration in the M- and MGN-treated groups and a decrease in the GO-treated group. Increase in SOD activity for all groups compared to the control. Increase in the GSH in the groups treated with M, GN, and GO and a decrease in the groups treated with MGN and MGO. Significant increase in the 8-OHdG marker in the M, GO, MGN, and MGO of the treated groups.
[42]	Day 8: DHES and tumor induction Day 12: treatment Day 14: end point	Colorectal carcinoma: − CT26-Luc: 1 × 10^6^ cells in Matrigel Pancreatic cancer: − PANC-1: 1 × 10^6^ cells in Matrigel	Gaseous plasma Administration: gas	Luminescence	No difference was found in tumor shrinkage by treatment with gas plasma (kINPen) supplemented with MoNoS adapters at 2 slm relative to argon gas-treated controls. However, at 5 slm, the kINPen treatment caused severe hemorrhage, while the adapters allowed this treatment at 5 slm, and tumor shrinkage was observed.
[43]	Day 6: DHES and tumor induction Day 10: treatment Day 14: end point	Squamous cell carcinoma of the head and neck: − SCC-15: 2 × 10^6^ cells in medium and Matrigel (1:1)	CDDP at 688 μM NAs-cisPT at 688 μM of CDDP and 24 μg Au and NAs 24 μg Administration: not specified	HE	Tumors treated with CDDP and NAs-cisPt showed significant tumor shrinkage, while those treated with NAs showed no significant change in tumor size. NAs-cisPt administration led to a deterioration of the CAM; however, tumors treated with CDDP and NAs had undamaged areas.
[44]	Day 3: DHES and tumor induction Days 11–13: treatment Day 14: end point	Neuroblastoma: − BE(2)C and IMR32 cells 2 × 10^6^ in medium	ATRA at 10 μM and 100 μM Administration: injection	PCR IHC	ATRA reduced cell proliferation and promoted a change in differentiation markers.
[45]	Day 10: DHES and tumor induction Day 10: treatment Day 12: end point	Prostate cancer: − PC-3: 5 × 10^5^ cells medium and Matrigel (1:1)	3α-diol at 10^−9^ M Administration: injection	-	-3α-diol can act as a neurosteroid in PCa cells to activate the GABAAR and may have a role in transforming androgen-dependent to growth factor-dependent pathways for CRPC progression.
[46]	Day 8: DHES, polyethylene ring, and tumor induction Days 9–13: treatment Day 14: end point	Cervical cancer: − SiHa: 1.5 × 10^5^ cells in medium and Matrigel (1:1)	AA at 0–20 mg/kg Administration: topical	-	Tumors induced in the CAM from the SiHa cervical cancer cell line treated with anisomelic acid had significantly lower growth than the controls.
[47]	Day 6: DHES and tumor induction Day 13: treatment Day 15: end point	Glioblastoma: − U87: 3–4 × 10^6^ cells in medium.	UDD at 500 μg/mL MW-RF at 500 μg/mL Administration: topical	HE PCR	Both types of nanoparticles were effective as they significantly reduced tumor size and angiogenesis. Furthermore, UDD and MW-RF reduced the expression of fibroblast growth factor 2 and vascular endothelial growth factor.
[48]	Day 7: DHES, silicon ring, and tumor induction Days 10 and 13: treatment Day 17: end point using 1 mL of 10% formalin for 60 min at room temperature	Colorectal carcinoma: − CT26 and HCT-116 Breast cancer: − 4T1 and MDA-MB-231 Glioblastoma: − U118MG and GL261 Hepatocellular carcinoma: − HepG2 Lung adenocarcinoma: − PC-9 and PC9/CR Prostate cancer: − LNCaP and PC-3 Melanoma: − A375 From all of them, 2 × 10^6^ cells in PBS and Matrigel (1:1)	CDDP at 0.2, 0.4, 1, 2, 4, and 10 mg/kg Sorafenib at 2 mg/kg Doxorubicin at 0.4 mg/kg Cyclophosphamide at 1, 10, or 100 mg/kg TMZ at 1 mg/kg 5-FU at 1 mg/kg Administration: topical	-	TMZ significantly reduced the weight of glioblastoma (U118MG, GL261) and melanoma tumor (A375). CDDP reduced the weight of PC-3, HCT-116, CT26, A375, and HepG2, but did not affect the weight of PC9/CR tumors. Doxorubicin significantly reduced the weight of mouse 4T1, PC-3, HCT-116, CT26, and HepG2 tumors. 5-FU reduced HCT-116 tumor weight. Sorafenib did not affect the weight of GL261 or A375, and it had a minor effect on HCT-116 tumor weight.
[49]	Day 3: DHES Day 6: tumor induction Day 10: treatment Day 17: end point	Ductal adenocarcinoma of the pancreas: − PDAC3 and SUIT2-028: 10^6^ cells in medium	CDDP at 213 μM USNP at 16.8 μg of Au in NAs-cisPt. Administration: topical The embryos with the combined treatments were subsequently irradiated with 4G and γ	HE	Tumors induced from the PDAC3 tumor line treated with NAs-cisPt significantly reduced their tumor volume. HE staining of SUIT2-028 tumors showed ductal structures typical of PDAC. Tumor weight after NAs-cisPt treatment did not differ from the untreated group; however, the addition of 4G and γ reduced the weight in both the untreated and NAs-cisPt groups.
[50]	Day 7: DHES, silicone ring, and tumor induction Day 8: treatment Day 14: end point	Oral Squamous Cell Carcinoma: − Cal-27: 1.5 × 10^6^ cells in medium and Matrigel (1:1)	CDDO-Me at 10 nM Administration: topical	HE IHC	CDDO-Me treatment significantly reduced tumor volume. There was no significant difference in Ki-67 expression between the two groups.
[51]	Day 4: DHES Day 10: tumor induction Treatment and end point day: not specified	Melanoma: − A375: 100,000 cells in medium	Ethanolic extract of olive leaves at 5, 15, and 100 μg/μL Administration: not specified	-	Doses of 15 µg/mL and 100 15 µg/mL of olive leaf extract showed a greater effect on cell growth and the development of narrow vessels in the A376 tumor.
[52]	DHES day: not specified Day 9: tumor induction Days 10, 12, 14, 15, and 17: treatment Day 18: end point	Breast cancer: − MDA-MB-231: 1 × 10^6^ cells in medium and Matrigel (1:1)	A-C2 at 20 mg/kg Anti-PD-L1 nanofitin (B11) at 20 mg/kg A-C2-B11 at 20 mg/kg Pembrolizumab at 2 mg/kg Administration: injection	-	Pembrolizumab, A-C2, and C2-B11 treatments significantly reduced tumor weight.
[53]	Day 10: DHES and tumor induction Day 16: treatment Day 17: end point	Neuroblastoma: − UKF-NB-4: 1 × 10^6^ in medium	CDDP at 100 µM Elli at 200 µM Administration: topical	qPCR IHC	CDDP and Elli reduced tumor weight compared to the control by 2 and 3.5 times, respectively. CD44 revealed that the primary tumor altered the upper epithelium and invaded the CAM. qPCR analysis of human alu showed that CDDP and Elli reduced the extravasation of tumor cells to the distant CAM and the liver, lungs, and brain.
[54]	Day 3: DHES Day 9: nylon ring and tumor induction Day 10: treatment Day 14: end point	Breast cancer: − MDA-MB-231: 7.5 × 10^5^ in Matrigel (1:1)	2e at 3 µM Administration: topical	HE	2e reduced tumor size compared to the control. HE showed the viability of the tumors in both conditions.
[55]	Day 4: DHES Day 10: plastic ring, tumor induction, and treatment Day14: end point	Breast cancer: − MCF-7 and MDA-MB-231: 10^5^ cells It does not specify when cells are suspended	Extracts of leaves of *Melissa officinalis:* − ethanolic extracts: MOE96 and MOE70 at 50 μg/mL − methanolic extract: MOM80 at 50 μg/mL RA at 50 μM UA at 50 μM Administration: topical	-	The compounds prevented tumor growth outside the ring. MOE96 showed a greater antiproliferative effect and antiangiogenic effects in the MCF7 line after 24 h of treatment, and in the MDA-MB-231 line after 96 h.
[56]	Day 9: DHES and tumor induction Days 11, 15, and 17: treatment Day 18: end point	Breast cancer: − MB-MDA-231: 1 × 10^6^ cells in medium	Doxorubicin at 50 μM Administration: topical	Metabolic and lipidomic profiling	Tumors treated with doxorubicin showed smaller sizes and weights. The metabolic and lipid analysis was different in the tumors treated with doxorubicin compared to the controls. Doxorubicin inhibits glycolysis, nucleotide synthesis, choline metabolism, and fatty acid metabolism. On the contrary, antioxidant pathways are activated.
[57]	Days 3 to 5: DHES Day 7: plastic ring and tumor induction Day 14: end point	Colorectal cancer: − MC-38: 0.5 × 10^6^ in Matrigel (1:1) Lung cancer: − A549: 0.5 × 10^6^ in Matrigel (1:1)	Air and carbogen Administration: through plastic tubes to the CAM	MRI (T1 and T2) HE IHC	MC-38 tumors showed volumes 65% larger than A549 tumors. A549 tumors showed significantly higher T2 values and no changes in T1 after exposure to carbogen, while MC-38 tumors showed no changes in T1 and T2. MC-38 tumors showed larger T1 and T2 in the center, while in A549 they were distributed on the surface. MCF-7 tumors showed a greater number of proliferative cells (Ki-67) distributed homogeneously. A549 tumors had fewer proliferative cells distributed irregularly on the surface. Based on HIF-1-α-positive cells, the density of hypoxic cells was higher for MC-38 tumors compared to A549.
[58]	Day 4: DHES Day 8: tumor induction Day 11: silicone ring and treatment Days 13 to 15: end point	Ovarian cancer: − SKOV-3: 2 × 10^6^ in Geltrex	CBD at 100 µM CBD-NP NP Administration: topical	HE	HE revealed that cells invaded the CAM to form the tumor mass. CBD and CBD-NP significantly reduced tumor growth by 1.38 and 1.5 times, respectively.
[59]	Day 10: DHES and tumor induction Day 13: treatment Day 15: end point	Pancreatic cancer: − MiaPaCa-2: 10^6^ in Matrigel and medium (75/25)	Doxorubicin at 184 nmol 8a at 184 nmol Administration: Intratumoral injections	HPLC	Doxorubicin and 8a reduced tumor size by 50%, and tumor necrosis was observed. HPLC revealed the same amount of doxorubicin in the tumors (30%), but 8a was not detected.
[60]	DHES day: not specified Day 8: tumor induction Day 10: treatment Day 13: end point	Breast cancer: − 4T1: 2 × 10^6^ cells in medium Matrigel (1:1)	Tetrocarcin-A at 2.5 µM Administration: topical	HE IHC	Tetrocarcin A reduced tumor growth compared to the control. Tetrocarcin A-treated tumors showed 60% of cleaved caspase 3 expression compared to the control (10%).
[61]	Day 3: DHES Day 9: treatment of cells and after tumor induction Day 16: end point	Ovarian cancer: − SKOV3: 1 × 10^6^ cells in Matrigel	ApoA1 at 100 μg/mL CDDP at 15 μM Treatment with cells	FC	The combination of ApoA1 and CDDP showed a greater reduction in tumor size than both treatments alone. In addition, the combination was synergistic.
[62]	Day 8: DHES Day 9: tumor induction with treated cells Day 14: end point	Colorectal cancer: − HCT116: 1.5 × 10^6^ treated cells in medium and Matrigel (1:1)	DZNep at 5 µM Treated cells prior to tumor induction	HE IHC	DZNep-treated tumors showed smaller sizes and fewer cells in clusters with large areas of Matrigel. However, DZNep-treated tumors also showed a greater capacity for CAM invasion and a higher degree of vascularization, displaying a more aggressive phenotype. DZNep reduced EZH2 expression levels in tumors. High heterogeneity of EZH2 and H3K27me3 staining intensity was detected in DZNep-treated tumors.

2e (3-(4-clorofenil)tieno[3,2-b]piridin-2-carboxilato de metilo); DZNep (3-deazaneplanocin A); 5-FU (5-fluorouracil); 8-OHdG (8-hydroxy-2′-deoxyguanosine); ALA (aminolevulinic acid); AA (anisomelic acid); ApoA1 (apolipoprotein A1); APE1 (apurinic/apyrimidinic endodeoxyribonuclease 1); 8a (arylboronate doxorubicin compound); CDDO-Me (bardoxolone-Methyl); CBD (cannabidiol); CBD-NPs (cannabidiol nanoparticles); CAM (chorioallantoic membrane); CDDP (cisplatin); NAs-cisPt (cisplatin comprised as prodrug in NAs); DHES (drill a hole in the eggshell); Elli (ellipticine); FC (fluorescence); PpIX (fluorescent protoporphyrin IX); GN (graphene); GO (graphene oxide); HE (hematoxylin and eosin); IHC (immunohistochemistry); M (melittin); MRI (magnetic resonance imaging); NAs (nano-architectures); NP (nanoparticles); NSCLC (non-small-cell lung cancer); PBS (phosphate-buffered saline); PtNPs (platinum nanoparticles); RA (rosmarinic acid); SOD (superoxide dismutase); TMZ (temozolomide); H3K27me3 (trimethylation of histone H3 lysine 27); TNBC (triple-negative breast cancer); USNP (ultra-small nanoparticles); UA (ursolic acid); VCR (vincristine); EZH2 (zeste homolog 2).

**Table 3:** The CAM assay as a methodology used for tumor induction ex ovo from cell lines.

Ref.	Methodology CAM Assay	Cell Line	Treatment	Applied Techniques	Results
[38]	The day of inoculation is not indicated. Drug treatment 72 h after inoculation. Euthanasia 24 h after treatment	Breast: − MDA-MB-231: 5 × 10^4^ cells per treatment	CDDP: 100 µg/mL, 5 µL (24 h) PtNP-10: 250 µg/mL, 5 µL (24 h) PtNP-40: 250 µg/mL, 5 µL (24 h)	Evaluation of tumor migration and localization in the CAM and embryo by CellTracker Green fluorescence. Tumor area quantification	Reduction in the tumor area in 24 h drug treatments, highlighting the reduction in PtNP-10 treatments. Reduction in CAM metastasis in PtNP treatments.
[53]	Day 3: TCE Day 10: tumor induction Day 13: treatment Day 14: euthanasia by incision in the vitelline arteries	Neuroblastoma: − Nbl UKF-NB-4: 5 × 10^4^ cells in 25 µL of serum-free medium	CDDP: 100 µM, 5 µL (24 h) Elli: 200 µL, 5 µL (24 h)	Cell tracking histology, immunohistochemistry, and qRT-PCR	A tumor was successfully induced from Nbl UFK-NB-4 cells in the CAM. CDDP and Elli treatments eliminated the intravasation and extravasation of UFK-NB-4 cells, as well as dramatically reduced the tumor size.
[63]	Day 3: TCE Day 7: tumor induction + treatment Day 11: euthanasia	Osteosarcoma: − U48484: 10^6^ cells in 50 µL of hydrogel added into a 3D scaffold Hepatocarciona: − HepG2, HB243, and HB282: 5 × 10^5^ cells in 50 µL of hydrogel added into a 3D scaffold	Osteosarcoma: BEZ235: 500 nM (72 h). drug mixed with cellular/hydrogel solution Hepatocarcinoma: Volasertib: 0.3 µM, 3 µM and 30 µM in PBS	Osteosarcoma: Histology and bioluminescence measurement Hepatocarcinoma: luciferase imaging study and cytotoxicity essay	Reduction by 20% of the U48484 fluorescence with BEZ235 treatment. Inhibition of 50% of the tumor growth of HB243 and HB282 treated with voasertib.
[64]	Day 3: TCE Day 9: engraftment of pre-treated cells Day 13: euthanasia	Osteosarcoma: − U2-OS and Saos-2: 10^6^ cells per treatment	15d-PGJ_2_: 20 µM (24 h). In vitro treatment before engraftment	Tumor area, morphology, and proliferation quantification Immunohistochemistry	Reduction in tumor size and density in U2-OS and Saos-2 lines previously treated. Reduction in the cell proliferation marker Ki-67 in the presence of the drug.
[65]	Day 3: TCE Day 10: tumor induction Day 13. euthanasia	Colorectal: − WiDr and HCT116: 5 × 10^5^ cells in 20 µL	Previous growth of cancer cells in Matrigel before graft	Histology, immunohistology, Western blot, and stem cell proteome array	Matrigel 3D pre-culture of the tumoral cells facilitates the vascularization of the CAM tumors and promotes differences in proliferation, protein markers, and gene expression among the different treatments.
[66]	Day 2 (after 54 h of incubation): TCE Day 7: tumor induction + treatment Day 12: euthanasia	Glioblastoma: − SF-8628: 1.3 × 10^5^ cells in 25 µL of PBS	Anti-IL13Rα2::PBD: 50 ng/mL, 500 ng/mL and 5 µg/mL in 25 µL of PBS; 29 min; in vitro treatment before cell graft	Luminescence measurement with luciferase	Dose–dependent antitumor effect of Anti-IL13Rα2 in the CAM tumor.
[67]	Day 3: transfer the contents of the egg into a sterile 6-well plate Day 7: tumor induction Day 8: treatment and euthanasia	Esophageal: TE1: spheroid implantation Incubation of 500 cells in 250 µL per well in 96-well plates. Three to five weeks incubation	Hyp Hyp:LDL 100:1 Hyp:LDL 200:1 79 µM in PBS with 0.17% DMSO (2 µg/ g of embryo) 6 h of incubation	CAM photo analysis using ImageJ software and spectroscopic measurement of fluorescence	Differences in proliferation, protein markers, and gene expression among the different treatments.
[68]	Day 3: TCE Day 9: tumor induction + treatment. Day 14: euthanasia	Myeloma: OPM-2: 3 × 10^5^ cells in 30 µL of mouse tail type 1 collagen solution in ×10 of DMEM and neutralized with 0.1 N NaOH	PIX: 1 µM	Fluorescence by the GFP labeling of cells and quantification by ELISA	Blockage of tumor growth in the presence of kinin due to a reduction in tumor area.
[69]	The TCE is not indicated. Day 6: tumor induction Day 10: first treatment Day 12: second treatment Day 15: euthanasia	Breast: MDA-MB-231: 2 × 10^6^ cells per CAM	Phemindole: 10 µL/mL	Amplification of the alu sequences of the tumor cells	Suppression of tumor formation with phemindole treatment at a dose of 10 uM.
[70]	Day 3: TCE Day 9: tumor induction Day 19: treatment Day 14: euthanasia	Human glioblastoma: U87: 4 × 10^5^ cells per CAM Canine melanoma: 17CM98: 4 × 10^5^ + 1/5 Matrigel per CAM Canine osteosarcoma: − D17: 6 × 10^5^ + 1/5 Matrigel	AVA: 10 mg/kg, intravenous injection; 50 µL CHC: 60 mg/kg, topical application around explant; 50 µL AZD3965: 2.5 µM per egg, intravenous injection; 50 µL AVA + CHC AVA + AZD	Tumor growth, tumor perfusion, and tumor hypoxia	Decrease in tumor size in the presence of the drugs, highlighting the effect of combined treatment in U87. Reduction in tumor perfusion in combined VPA + CHC therapy. Reduction in hypoxia in VPA treatment.
[71]	The TCE is not indicated. Day 9 or 10: tumor induction Day 11 or 12 (48 h after induction): treatment Day 16: euthanasia by decapitation	Glioma tissue: 1–2 mm diameter piece, with Matrigel Glioblastoma: U-87: 0.75 × 10^6^ MG: 0.75 × 10^6^ LN-18: 1 × 10^6^	Carboplatin: 8 mg/kg (48 h) TMZ: 4 mg/kg (48 h)	Histology and immunostaining, genomic analysis, and drug sensibility assays	Tumor-forming capacity in the CAM. Antitumor effect of drugs on tumors formed in the CAM.

Anti-IL13Rα2 (anti-interleukin 13 receptor subunit alpha 2); AVA (avastin); AZD (AZD3965); CAM (chorioallantoic membrane); CDDP (cispatin); CHC (α-cyano-4-hydroxycinnamic acid); DMEM (Dulbecco’s Modified Eagle Medium); DMSO (dimethyl sulfoxide); D17 (canine osteosarcoma cell line); Elli (ellipticine); HB243 (hepatoblastoma cell line); HB282 (hepatoblastoma cell line); HCT116 (human colorectal cell line); HepG2 (human liver cancer cell line); Hyp (hypericin); LDL (low-density lipoprotein); LN-18 (human glioblastoma cell line); MCF-7 (human breast cancer cell line); MDA-MB-231 (human breast cancer cell line); MG (human glioblastoma cell line); Nbl UKF-NB-4 (advanced neuroblastoma cells); PFA (paraformaldehyde); PIX (pixantrone); PtNPs-10 (platinum nanoparticles coated with PVP, molecular weight of 10.000); PtNPs-10 (platinum nanoparticles coated with PVP, molecular weight of 40.000); OPM-2 (human myelome cell line); Saos-2 (human origin osteosarcoma cell line); SF-8628 (human glioblastoma cell line); TCE (transfer of egg contents into a sterile container); TE1 (human esophageal squamous cell carcinoma); TMZ (temozolomide); U2-0S (human origin osteosarcoma cell line); U48484 (transgenic rhabdomyosarcoma cell line); U87 (human glioblastoma cell line); WiDR (human colorectal adenocarcinoma cell line); 15d-PGJ_2_ (15-Deoxy-∆^12,14^-prostaglandin J_2_); 17CM98 (canine oral melanoma cell line).

**Table 4:** The CAM assay as a methodology used to carry out a study on the formation of metastases.

Ref.	CAM Assay Methodology	Cell Line	Techniques	Results
[53]	Day 10: DHES and tumor induction Day 16: treatment with CDDP (100 µM) or Elli (200 µM) topically Day 17: euthanasia	Bone marrow metastases that are high risk: − UKF-NB-4: 1 × 10^6^ cells in medium	Intravasated/extravasated cells; qPCR after DNA extraction for *Alu* sequences	CDDP and ELLI exhibited significant inhibitory activity against extravasation to the liver, lungs brain, and distal CAM.
[62]	Day 8: DHES Day 9: tumor induction Day 14: euthanasia	Colorectal cancer: − HCT116: 1.5 × 10^6^ cells in Matrigel − HCT116 DZNep-treated:1.5 × 10^6^ cells in Matrigel (1:1)	IHC of CAM tumors FFPE; relative vessel density intra- and peri-tumoral	Tumors without EZH2 expression have an increase in the number of vessels and higher tumor aggressiveness.
[72]	Day 8: DHES and tumor induction placed in a polypropylene ring Day 16: euthanasia	Pancreatic cancer: − PANC-1: 1–3 × 10^6^ cells in Matrigel	PCR after DNA extraction from chicken liver	Chicken liver cells have a high expression of CK7.
[73]	Day 7: DHES and tumor induction Day 14: euthanasia	Neuroblastoma: − GFP-labeled SK-N-AS in hypoxic: 2 × 10^6^ cells in medium	MRI; frozen tissue slices analyzed with an epi-fluorescent microscope	MRI technique can detect metastasis deposits of up to 12 cells.
	Day 7: DHES and tumor injection in the chicken brain Day 14: euthanasia	Neuroblastoma: − GFP MPIO-labeled SK-N-AS: 3 × 10^6^ cells in medium		
[74]	Day 10: DHES and tumor induction placed in a plastic ring Day 18: anesthesia by ice and euthanasia	Colorectal cancer: − LS174T: 1 × 10^5^ cells in medium − LS17AT LNA-anti-miR-21:1 × 10^5^ cells in medium	qPCR after DNA extraction for *Alu* sequences; transfection	Chicken liver cells have a significantly decreased number of metastases than LNA-anti-miR-21 groups.
[75]	Day 3: DHES Day 10: tumor induction Day 11: treatment Day 17: euthanasia	Prostate cancer: − LNCaP and IGR-CaP1 Lung cancer: − A549 and H1299 − 1 × 10^3^ cells in Matrigel (1:1)	Fluorescent macroscopy imaging; 3D chick embryo for fluorescence detection; FFPE histochemistry	CDDP and docetaxel treatment decreased the metastatic foci detection.

CAM: chorioallantoic membrane; CDDP: cisplatin; DNA: deoxyribonucleic acid; DHES: drill a hole in the eggshell; DZNep: EZH2 inhibitor; ELLI: ellipticine; FFPE: formalin-fixed paraffin-embedded; GFP: green fluorescent protein; IHC: immunohistochemistry; miR: micro-RNA; LNA: locked nucleic acid; MRI: magnetic resonance imaging; MPIO: micron-sized iron particles; qPCR: real-time polymerase chain reaction.

**Table 5:** The CAM assay as a patient-derived xenograft model.

Ref.	Sample	CAM Xenograft Model	Applied Studies	Results
[71]	Biopsy from 60 patients with glioma undergoing treatment	Ex ovo Days 8–10: freshly resected tumor implanted (1–2 mm) Two days after implantation: treatment injected into the CAM vasculature CB: 8 mg/Kg and TMZ: 4 mg/Kg Day 16: euthanasia by decapitation	Genomic analysis HE IHC	A total of 98.3% of glioma specimens established xenograft tumors on the CAM. The glioma CAM-PDX model retained the histopathology and molecular characteristics of the original tumor. Higher CAM-PDX tumorigenicity is associated with poorer prognosis in glioma patients.
[76]	Isolate CSC from 4 patients with CRC and BM.	In ovo Day 9: DHES and implant 3 × 10^6^ cells Day 18: remove the tumor of the CAM, wash with PBS, and transfer in PFA for 48 h	PCR	Tumor formation was correctly established from the BM-SC-CRC lines. In addition, they acquired invasion and migration capabilities in the CAM. The cell lines from patients 1 and 2 were capable of generating metastasis.
[77]	Biopsy of CM (Stage IV) and RB from 2 patients undergoing enucleation	In ovo Day 9: the air sac was punctured and suctioned. DHES and place a sterile silicone o-ring on a visible vascularization area Day 10: three PDX were implanted in the o-ring Day 17: euthanasia by hypothermia	HE IHC	RBs and CM PDXs successfully induced tumors in the CAM. Furthermore, angiogenesis was observed in the tumor and intratumoral periphery.
[78]	Biopsy from 6 advanced-stage uveal melanoma patients	In ovo Day 5: remove 4–10 mL albumin Day 6: DHES Day 7: lacerate the surface of the CAM causing bleeding. Three methods of implantation: Engrafting the tumor sample with Matrigel and a plastic ring Only with Matrigel Native Day 8: ring removed in group 1 Day 18: euthanasia by decapitation	Ultrasound scans Optical tomography Fluorescein angiography HE IHC	Biopsy fragments from patients with uveal melanoma were successfully established in the CAM. Furthermore, the techniques applied in their study allowed the CAM assay to be used as a PDX model in experimental oncology.
[79]	CTC of 35 cancer patients (6 prostate, 6 breast, 23 lung)	In ovo Day 9: DHES and place the CTC suspension onto the CAM Day 18: euthanasia	HE IHC PCR NGS analysis	Tumors from biopsies grafted onto the CAM showed genomic concordance with the original patient’s tumor and its liquid biopsy by scanning the DNA sequence using NGS. Furthermore, these results generated a patent: “WO2020/089560A1; 7 May 2020.”
[80]	CCSC of 10 women diagnosed with breast cancer in different stages of the disease	In ovo Day 4: DHES Day 8: Engraft tumorospheres of CCSC in Matrigel directly. Day 16: Euthanasia by decapitation.	HE	Histological studies showed that tumors in the CAM maintained the initial structure. Furthermore, biopsies from patients with a high Ki-67 index were the most likely to develop tumors in the CAM membrane.
[81]	LM of CR adenocarcinoma biopsy	In ovo Day 3: open DHES Day 8: apply silicone ring and implant the suspension of tumor cells derived from CRLM Day 12: xenograft was transferred to the membrane of another egg Day 16: euthanasia	IHC	Tumor induction in the CAM was successful, generating solid tumors with increased angiogenesis around them from the vascularization of the CAM. Likewise, there was a concordance between the response of the patients and that observed in the tumors in the CAM in terms of aggressiveness and metastasis formation.
[82]	Isolate cells from the patient’s ccRCC	Day 7: DHES Day 10: patient tissue-derived primary cancer was implanted cells in Matrigel (2–3 mm) and isolated 2 × 10^6^ cells in Matrigel Day 20: euthanasia by being placed on ice for 20 min	Histology	A high efficiency was obtained in the grafting of PDX in the CAM since it was generated in just 10 days, and it could be a very promising assay to carry out studies on drug detection in the tumor of an individual patient.
[83]	MTCs from 2 patients	Ex ovo Day 3: eggshell crack Day 10: silicon ring and implement tumor tissue (2 mm) Day 16: histological analysis	Histology IHC	The samples grafted into the CAM remained alive and also expressed specific neuroendocrine markers synaptophysin and chromogranin A.
[84]	Biopsy from 9 patients with ovarian cancer	In ovo Day 4: DHES Day 10: a silicone ring and an ovarian piece were placed into the ring Day 15: survival rate evaluated	Histology IHC	The survival rate of chicken embryos was 97.2%. After tumor induction, there was an increase in angiogenesis around the tumor.
[85]	Biopsy from 24 ccRCC patients	In ovo Days 8–10: tumor fragment implanted onto the CAM in Matrigel. 2 days after implantation: treatment with Sunitinib IV. Day 16: euthanasia	HE IHC	It was observed macroscopically that after tumor induction in the CAM, the number of blood vessels around it increased. CAM tumor xenografts from patients with ccRCC retain the histopathological characteristics of the original tumor.
[86]	Biopsy of 22 patient with bladder cancer	In ovo Day 7: DHES and place a silicone ring. After 2 h, engraft homogenates from cryopreserved or engraft fresh tumors in Matrigel Day 11: daily treatment with saline and/or DMSO vehicle control; GMZ; cisplatin; afatinib; abemaciclib; AZD4547 Day 18: euthanasia	HE IHC	Primary tumors grafted onto the CAM as ground homogenates adopted a different morphological phenotype compared to the original tumors. Fresh tumors grew well and showed similar histology to the original tumor. Ki-67 expression was better retained in tumors grafted from frozen samples compared to fresh ones. Clinical resistance to cisplatin-based chemotherapy of pre-NAC MIBC tumors was maintained in CAM-PDX.
[1]	Three ccRCC patients’ tumor tissue	In ovo Day 9: patients’ tumor fragments with Matrigel Days 10–16: treatment with vehicle or sorafenib Day 16: eggs sacrificed by decapitation	qPCR IHC HE	The treatment of tumors induced in the CAM from patient biopsies showed the same response as patients in the clinic. The xenograft from patient 1 responded, while the xenografts derived from patients 2 and 3 did not respond.
[87]	Fresh tumor samples from ovarian cancer patients	In ovo Day 10: DHES and a Teflon ring. Inoculation of tumor Day 13: 0.1 mL of nanoparticle or nanoparticle/doxorubicin solution was injected into the CAM blood vessel Day 19: sacrifice eggs	HE	PMO loaded with doxorubicin decreased tumor volume. All eggs survived the injection of nanoparticles with doses of up to 200 μg of doxorubicin. The internal organs (liver, heart, intestines, kidneys, and spleen) appeared normal. Macroscopically, the tumor was observed four days after transplantation, and the size of the tumor grew exponentially six days later.

BM (brain metastasis); BM-SC-CRC (brain metastasis stem cell lines from patients with colorectal cancer); CAM (chick embryo chorioallantoic membrane); CB (carboplatin); ccRCC (clear cell renal cell carcinoma); CCSCs (circulating cancer stem cells); CM (choroidal melanoma); CR (colorectal cancer); CSCs (cancer stem cells); CTCs (circulating cancer biomarkers), DHES: drill a hole in the eggshell; HE (hematoxylin and eosin); IHC (immunohistochemistry); IV (intravenous); LM (liver metastasis); MTC (medullary thyroid cancer); PDX (tissue segment of each tumor tissue); RB (retinoblastoma); TMZ (temozolamide).

## Data Availability

The data presented in this study are available in the main article.
